# Phenolic-Rich *Aronia melanocarpa* (cv. Nero) Extract Attenuates Doxorubicin-Induced Nephrotoxicity Associated with Reduced Oxidative Stress, Inflammation, and Endoplasmic Reticulum Stress

**DOI:** 10.3390/molecules31203732

**Published:** 2026-10-17

**Authors:** Meltem Arıkan Malkoç, Serap Özer Yaman, Hüseyin Çınkılıç, Esin Yuluğ, Yakup Kara, Birgül Kural, Sevgi Kolaylı

**Affiliations:** 1 Vocational School of Health Sciences, Karadeniz Technical University, Trabzon 61080, Türkiye; 2 Department of Medical Biochemistry, Trabzon University, Trabzon 61080, Türkiye; serapozeryaman@trabzon.edu.tr; 3 Department of Histology and Embryology, Faculty of Medicine, Karadeniz Technical University, Trabzon 61080, Türkiye; huseyinedip96@gmail.com (H.Ç.); eyulug@ktu.edu.tr (E.Y.); 4 Department of Chemistry, Faculty of Science, Karadeniz Technical University, Trabzon 61080, Türkiye; yakupkara@ktu.edu.tr; 5 Department of Medical Biochemistry, Faculty of Medicine, Karadeniz Technical University, Trabzon 61080, Türkiye; bvanizorkural@ktu.edu.tr; 6 Department of Biochemistry, Faculty of Medicine, Nakhchivan State University, Nakhchivan AZ7000, Azerbaijan

**Keywords:** *A. melanocarpa*, antioxidant, nephrotoxicity, endoplasmic reticulum stress

## Abstract

*Aronia melanocarpa* (black chokeberry) is a polyphenol-rich berry recognized as a functional food because of its potent antioxidant properties. This study investigated the phenolic composition, antioxidant capacity, and nephroprotective effects of *A. melanocarpa* extract (AME) against doxorubicin (DXR)-induced renal injury. Antioxidant activity was evaluated by determining total phenolic content (TPC), total flavonoid content (TFC), ferric reducing antioxidant power (FRAP), and 2,2-diphenyl-1-picrylhydrazyl (DPPH) radical-scavenging activity. Thirty-six female Sprague–Dawley rats (180–220 g) were randomly allocated to four groups (*n* = 9 per group): Control, AME, DXR, and AME + DXR. AME (200 mg/kg/day) was administered orally for 21 consecutive days as a pretreatment, and acute nephrotoxicity was induced by a single intraperitoneal injection of DXR (20 mg/kg) on day 21. Samples were collected 24 h after DXR administration. Renal injury was evaluated using biochemical, histopathological, oxidative stress (OS), inflammatory, endoplasmic reticulum stress (ERS), and apoptosis-related parameters, together with TUNEL staining. HPLC–PDA analysis of 29 targeted phenolic compounds identified cya-nidin-3-galactoside (2440 µg/g FW), cyanidin-3-arabinoside (350 µg/g FW), chlorogenic acid (251.83 µg/g FW), and rutin (32.29 µg/g FW) as the major constituents. DXR markedly impaired renal function and increased oxidative stress, ERS, inflammation, and apoptosis, whereas AME pretreatment significantly attenuated these alterations. These findings are consistent with a protective effect of phenolic-rich AME against acute DXR-induced renal injury in rats, associated with attenuation of oxidative stress, inflammation, ERS, and apoptosis. Further studies are needed to confirm these findings and evaluate their potential clinical relevance.

## 1. Introduction

*Aronia melanocarpa* (Michx.) Elliott cv. ‘Nero’, commonly known as black chokeberry, is a polyphenol-rich species belonging to the Rosaceae family that has attracted significant interest as a functional food [1,2]. Its fruits are widely consumed as juices, syrups, jams, teas, wines, and dietary supplements and are particularly rich in anthocyanins, proanthocyanidins, flavonoids, vitamins, and trace elements [3]. These bioactive constituents have been associated with antioxidant, anti-inflammatory, antimicrobial, anticancer, antidiabetic, and immunomodulatory activities [4,5]. Among the major anthocyanins in *A. melanocarpa*, cyanidin-3-O-galactoside (C3Ga) is particularly abundant and exhibits potent antioxidant and radical-scavenging properties [6].

Doxorubicin (DXR) is a widely used chemotherapeutic agent whose clinical efficacy is constrained by dose-dependent nephrotoxicity [7]. The pathogenesis of DXR-induced renal damage involves excessive reactive oxygen species (ROS) generation, impaired endogenous antioxidant defenses, persistent inflammation, and apoptosis [1,8]. Continuous oxidative and inflammatory stress disrupts cellular homeostasis and exceeds protein folding capacity, leading to endoplasmic reticulum (ER) dysfunction and activating the unfolded protein response (UPR) [9]. While transient ER stress serves an adaptive protective role, sustained UPR activation drives pro-apoptotic pathways [10]. Glucose regulatory protein 78 (GRP78) functions as a key regulator of ER homeostasis, and its dissociation from ER stress sensors triggers UPR signaling pathways mediated by inositol-requiring enzyme-1 alpha (IRE1α), PKR-like ER kinase (PERK), and activating transcription factor-6 (ATF6) [11]. In particular, prolonged activation of the IRE1α–CHOP axis exacerbates renal injury by upregulating pro-apoptotic markers such as C/EBP homologous protein (CHOP) and caspase-3 [12]. Nuclear factor erythroid 2-related factor 2 (Nrf2) is a crucial transcription factor involved in regulating the gene expression of antioxidant proteins and maintaining ER redox homeostasis [13]. Thus, concurrent assessment of Nrf2 and ER stress-related biomarkers may provide insight into the relationship between redox imbalance and ER stress during DXR-induced renal injury.

Natural products rich in polyphenols have attracted increasing interest as potential protective agents against renal injury. Previous experimental studies have demonstrated the renoprotective potential of *A. melanocarpa* and its bioactive constituents across various models of kidney dysfunction. Specifically, chokeberry extract was reported to attenuate cisplatin-induced renal oxidative stress and preserve antioxidant capacity [14], while Aronia-derived anthocyanins reduced renal dysfunction, oxidative stress, and inflammatory responses in a renal ischemia–reperfusion model [15]. Furthermore, Smereczański et al. [16] demonstrated that *A. melanocarpa* berry extract protected against functional, biochemical, and histopathological alterations in chronic cadmium-induced nephrotoxicity. Collectively, these findings support the broad renoprotective potential of *A. melanocarpa*. However, the potential association of AME-mediated renal protection with oxidative stress, inflammation, Nrf2 signaling, ER stress-related biomarkers, and apoptosis has not been comprehensively evaluated in this model. Therefore, the novelty of the present study lies in integrating a detailed phenolic characterization of AME with comprehensive biochemical and histopathological assessments, alongside the targeted evaluation of oxidative, inflammatory, ER stress, and apoptotic pathways in an acute rat model of DXR-induced nephrotoxicity. We hypothesized that AME pretreatment would attenuate DXR-induced renal injury, and that this protection would be associated with restored redox homeostasis and attenuated inflammatory, ER stress-related, and apoptotic responses. Accordingly, this study aimed to characterize the phenolic profile and antioxidant capacity of AME and to evaluate its protective efficacy against DXR-induced nephrotoxicity across these interconnected cellular stress and histopathological markers.

## 2. Results and Discussion

### 2.1. Phenolic and Antioxidant Properties of AME

The phenolic composition and in vitro antioxidant properties of the analyzed *A*. *melanocarpa* berries and their ethanolic extract are presented in Table 1. TPC of the fresh aronia berries was determined to be 15.75 ± 1.04 mg GAE/g FW, corresponding to a concentration of 3.11 ± 0.21 mg GAE/mL in the ethanolic extract. Similarly, the TFC was measured as 1.16 ± 0.03 mg QE/g FW in the raw fruit and 0.23 ± 0.01 mg QE/mL in the liquid extract. The in vitro antioxidant capacity was assessed using FRAP (37.95 ± 0.61 µmol FeSO4·7H_2_O/mL extract) and DPPH radical scavenging assays. The ethanolic extract exhibited notable free radical scavenging activity, with a DPPH SC50 value of 0.23 ± 0.04 mg/mL (Table 1).

The measured TPC and TFC values reflect a rich polyphenolic profile in the prepared ethanolic extract. In the literature, reported TPC values for aronia berries evaluated on a fresh weight basis range between 13 and 17 mg GAE/g FW [17,18], which is in close agreement with our current finding (15.75 ± 1.04 mg GAE/g FW). Although some previous studies reported higher TPC values on a dry weight basis (e.g., ~30 mg GAE/g DW) [19], direct numeric comparisons across different weight bases should be interpreted with caution due to the substantial contribution of moisture content in fresh berry tissues. These variations across studies can be primarily attributed to differences in sample state (fresh vs. dry weight), extraction solvent polarity, aronia cultivar, geographical origin, and harvest time [17]. Although methanol is widely recognized as an efficient extraction solvent, diluted ethanol was deliberately selected in the present study to ensure safety for subsequent in vivo experimental applications. Furthermore, the markedly lower TFC relative to TPC indicates that non-flavonoid phenolic compounds, particularly anthocyanins and phenolic acid derivatives, constitute a substantial proportion of total phenolics [18]. Regarding antioxidant capacity, the combination of FRAP reducing power and DPPH SC50 values is highly consistent with previously published data for aronia extracts (e.g., SC50 of 0.29 ± 0.00 mg/mL), corroborating the potent antioxidant capacity of *A. melanocarpa* compared to other natural polyphenol sources [20].

In the present study, the phenolic profile of the ethanolic AME was comprehensively characterized using HPLC-PDA. Qualitative and quantitative determinations were performed targeting 29 distinct compounds via two specialized chromatographic runs. Specifically, 26 phenolic acids and flavonoids were separated, with the corresponding standard chromatogram provided in Figure S1 and its detailed analytical validation parameters previously established by Kara et al. [21]. In addition, three major anthocyanins (cyanidin-3-galactoside, cyanidin-3-glucoside, and cyanidin-3-arabinoside) were quantified under anthocyanin-specific elution conditions; their representative chromatogram alongside respective LOD and LOQ values is presented in Figure S2. The results indicated that chlorogenic acid was the predominant phenolic compound, whereas rutin, protocatechuic acid, and quercetin were present at moderate concentrations. In contrast, chrysin, pinocembrin, caffeic acid, p-coumaric acid, and trans-cinnamic acid were detected only in trace amounts.

Among the analyzed anthocyanins, cyanidin-3-galactoside (C3Ga) was identified as the predominant anthocyanin and the most abundant phenolic compound, whereas cyanidin-3-arabinoside (C3A) was detected at lower levels. Cyanidin-3-glucoside was not detected because its concentration was below the LOD. Consistent with previous reports, chlorogenic acid was the predominant phenolic acid identified in AME, while C3Ga represented the major phenolic compound overall [17,22]. Both C3Ga and chlorogenic acid have been extensively reported to possess antioxidant, anti-inflammatory, and antitumor activities, thereby contributing to the biological potential of aronia-derived products [23,24].

### 2.2. Histopathological and Biochemical Findings

**Figure 1:**
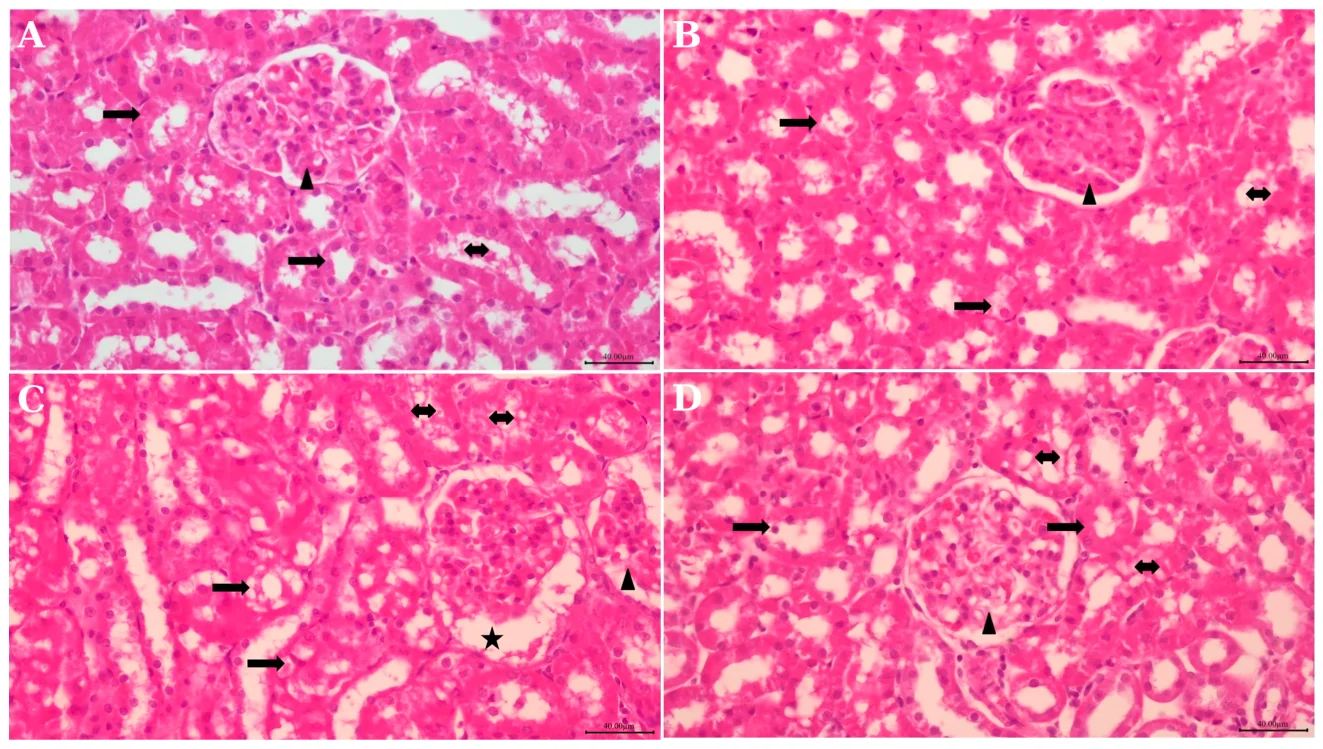
Photomicrographs of rat kidney tissues (H&E, ×400). (**A**) Control group: Normal renal histological architecture with well-preserved glomeruli (arrowheads), intact tubular epithelial cells (arrows), and minimal intratubular hyaline accumulation (double-headed arrows). (**B**) AME group: Histological appearance comparable to the control group, showing well-preserved glomeruli (arrowheads), intact tubular epithelial cells (arrows), and minimal intratubular hyaline accumulation (double-headed arrows). (**C**) DXR group: Degenerated tubular epithelial cells (arrows), dilatation of Bowman’s space (stars), shrunken glomeruli (arrowheads), and increased intratubular hyaline accumulation (double-headed arrows). (**D**) AME + DXR group: Marked improvement in renal histological architecture, with preservation of glomerular structures (arrowheads) and tubular structures, reduced tubular epithelial degeneration (arrows), and decreased intratubular hyaline accumulation (double-headed arrows). Photomicrographs of rat kidney tissues (H&E, ×400). H&E: Hematoxylin and eosin; AME: Aronia melanocarpa extract; DXR: Doxorubicin.

Histopathological findings are presented in Figure 1 and Table 2. Hematoxylin and eosin (H&E) staining revealed a normal renal architecture in both the control and AME groups, characterized by intact glomeruli and well-preserved tubular structures (Figure 1A,B). In contrast, the DXR-treated group exhibited marked structural alterations compared with the control and AME groups, including prominent widening of the Bowman’s space, degeneration of glomerular and tubular epithelial cells, and intratubular hyaline accumulation (*p* < 0.05) (Table 2, Figure 1C). Although intertubular vascular congestion appeared increased in the DXR group relative to the control group, this change did not reach statistical significance (*p* > 0.05). Notably, co-administration of AME with DXR markedly attenuated these histopathological alterations. The AME-treated group displayed significant reductions in glomerular and tubular epithelial degeneration, Bowman’s space dilation, intertubular inflammatory cell infiltration, and intratubular hyaline deposition compared with the DXR group (*p* < 0.05) (Table 2, Figure 1D).

**Figure 2:**
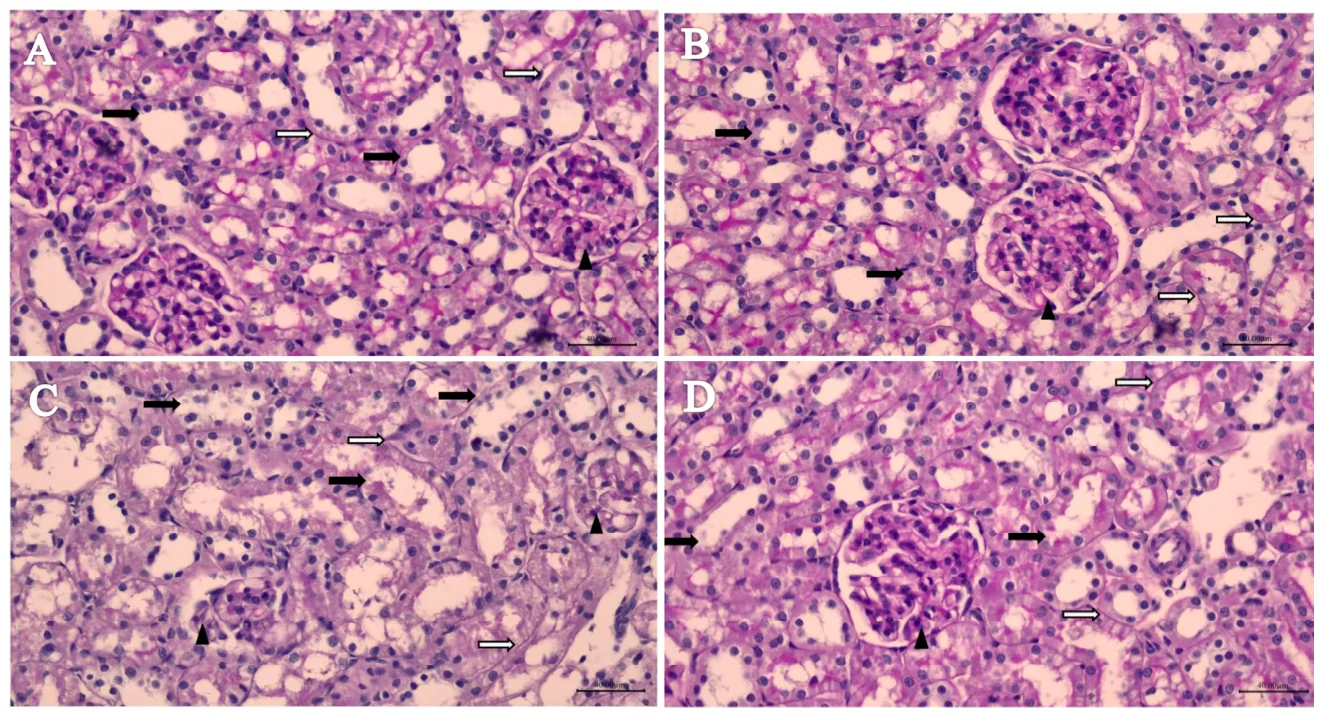
Histological appearance of kidney tissues stained with PAS (PAS, ×400). (**A**) Control group: Normal renal histological architecture with intact tubular epithelial cells (black arrows), well-preserved glomeruli (arrowheads), and strong PAS-positive staining of the basement membranes (white arrows). (**B**) AME group: Renal histology was comparable to that of the control group, with preserved tubular epithelial cells (black arrows), intact glomeruli (arrowheads), and strong PAS-positive basement membrane staining (white arrows). (**C**) DXR group: Tubular epithelial cells (black arrows) exhibited marked degeneration, accompanied by shrunken glomeruli (arrowheads) and a pronounced reduction in PAS-positive staining of the basement membranes (white arrows), indicating basement membrane injury. (**D**) AME + DXR group: Renal tissue showed marked histological improvement, characterized by preservation of tubular epithelial cells (black arrows), intact glomeruli (arrowheads), and markedly improved PAS-positive basement membrane staining (white arrows) relative to the DXR group, although not fully restored.

Periodic acid–Schiff (PAS) staining further supported these findings. Kidney sections from the Control and AME groups exhibited normal histological features with strong PAS-positive staining in glomeruli, tubular epithelial cells, and basement membranes (Figure 2A,B). In contrast, kidney sections from the DXR group demonstrated pronounced tubular epithelial degeneration accompanied by a marked reduction in PAS-positive basement membrane staining, indicating structural damage (Figure 2C). However, concurrent administration of AME substantially preserved PAS reactivity and renal morphology, demonstrating a partial restoration of renal histological structure compared with the DXR group (Figure 2D).

The histopathological findings were consistent with the biochemical indicators of renal injury (Table 3). DXR administration induced significant renal dysfunction, as demonstrated by marked increases in serum creatinine and blood urea nitrogen levels, together with elevated kidney injury molecule-1 and neutrophil gelatinase-associated lipocalin levels. These changes indicate impaired renal function and pronounced tubular injury. Although KIM-1, NGAL, BUN, and CRE levels were numerically higher in the AME-only group than in the control group, none of these differences reached statistical significance, and histopathological examination revealed no marked renal injury, indicating that AME was well tolerated at the tested dose. As described in the Methods, a dedicated vehicle control group was not included, and a minor influence of the solvent on these values therefore cannot be excluded; however, these elevations cannot be interpreted as evidence of AME-induced renal damage, as they cannot be reliably distinguished from inter-individual biological variability or minor physiological effects of the ethanolic vehicle.

The nephrotoxic effects of DXR are largely attributed to excessive ROS generation, and the histomorphological alterations observed in the present study are consistent with previously reported features of DXR-induced nephrotoxicity [25,26,27]. AME pretreatment significantly attenuated DXR-induced renal injury, as reflected by reductions in CRE, BUN, KIM-1, and NGAL levels compared with the DXR group (*p* < 0.001). However, these parameters remained significantly different from those of the control group, indicating partial protection rather than complete restoration of renal function and injury markers. This protective effect may be associated, at least in part, with the antioxidant properties of AME and its potential to limit ROS-mediated oxidative damage [28]. Consistent with these biochemical findings, histopathological improvement was evident in the AME + DXR group, although residual alterations remained compared with the control group. Future studies employing a dedicated vehicle control group or an ethanol-free formulation of AME would allow the contribution of the solvent to be addressed directly.

#### 2.2.1. Effects of AME on Oxidative Stress and Inflammation

**Figure 3:**
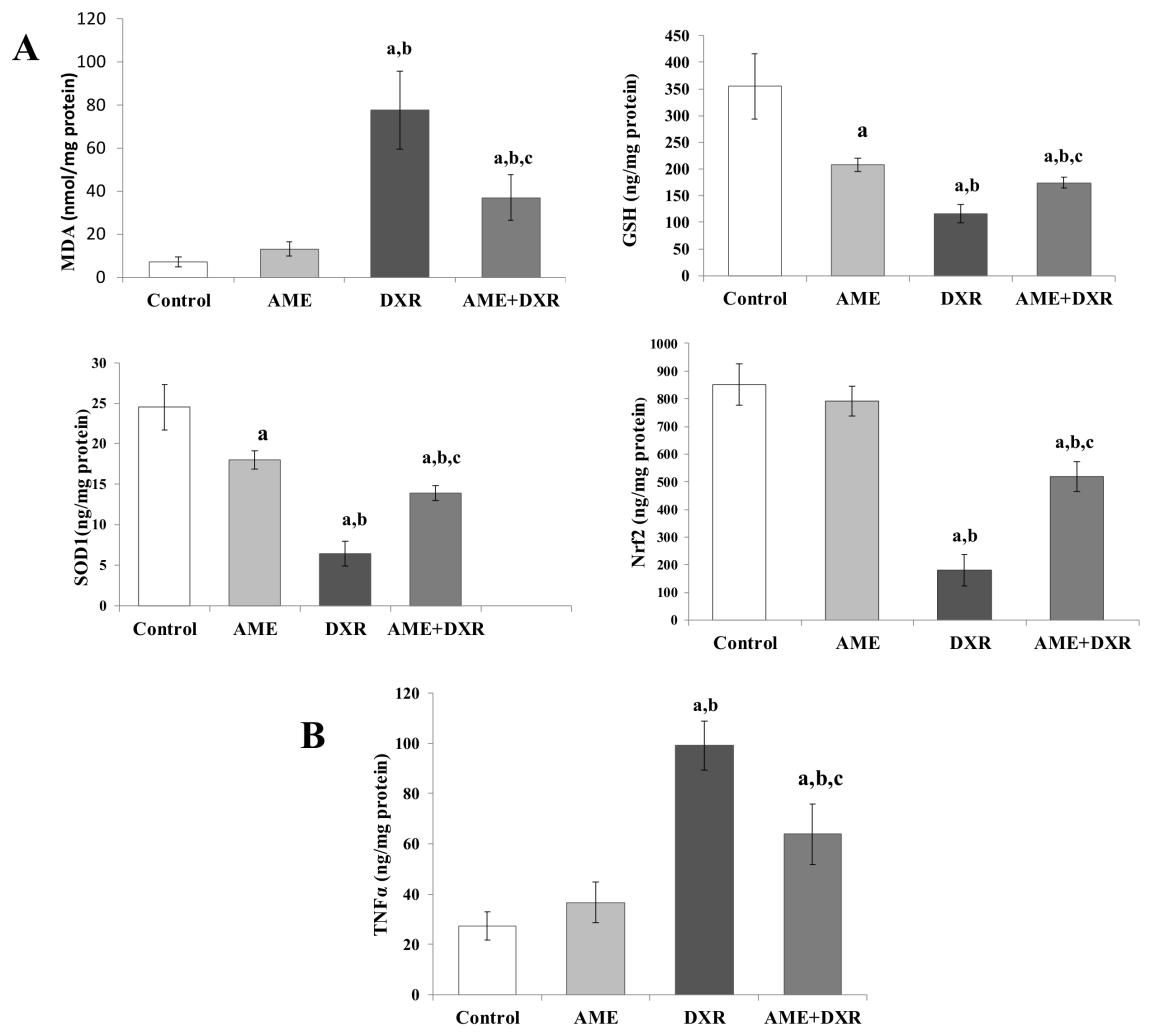
Oxidative stress (**A**) and inflammatory (**B**) parameters in kidney tissues of the experimental groups. (**A**) Malondialdehyde (MDA), nuclear factor erythroid 2-related factor 2 (Nrf2), glutathione (GSH), and superoxide dismutase 1 (SOD1) levels. (**B**) Tumor necrosis factor-alpha (TNF-α) levels. Data are expressed as mean ± standard deviation (SD) (*n* = 9). Statistical analysis was performed using one-way analysis of variance (ANOVA) followed by Tukey’s post hoc test. Differences were considered statistically significant at *p* < 0.05. a: significantly different from the Control group; b: significantly different from the AME group; c: significantly different from the DXR group. AME: *Aronia melanocarpa* extract; DXR: Doxorubicin.

Given that redox imbalance is widely recognized as a major mechanism underlying DXR-induced renal injury, the present study evaluated the protective effects of AME against oxidative stress and inflammation. As illustrated in Figure 3A,B, DXR administration resulted in marked oxidative damage, evidenced by significantly elevated renal malondialdehyde (MDA) and tumor necrosis factor alpha (TNF-α) levels, accompanied by profoundly reduced levels of glutathione (GSH), superoxide dismutase (SOD1) and Nrf2 (*p* < 0.001). In contrast, pretreatment with AME markedly improved the redox status, as reflected by reduced MDA and TNF-α levels and significantly increased GSH, SOD1, and Nrf2 levels compared with the DXR group (*p* < 0.001) (Figure 3).

Oxidative stress is widely recognized as a major contributor to DXR-induced nephrotoxicity, and reduced Nrf2 signaling has been associated with impaired antioxidant defense in injured kidneys [29]. Consistent with this, AME pretreatment was associated with increased renal Nrf2, GSH, and SOD1 levels together with reduced MDA levels [30]. The phenolic composition of AME may have contributed to these effects; notably, C3Ga, the predominant anthocyanin of AME, has been reported to activate the Nrf2/ARE signaling pathway in other experimental settings, which is consistent with the higher Nrf2 levels observed here [31,32]. However, because AME was administered as a complex extract, the individual contribution of C3Ga or any other constituent to the observed effects cannot be determined from the present data, and a causal role for Nrf2 activation cannot be established on the basis of these biomarker measurements.

With regard to inflammation, DXR administration markedly increased renal TNF-α levels in the present study, whereas AME pretreatment significantly reduced them (Figure 3B). Although NF-κB signaling was not assessed here, the observed changes in TNF-α are consistent with the anti-inflammatory activities reported for anthocyanin-rich polyphenols in previous studies [14,26]. Collectively, these findings suggest that AME pretreatment attenuated DXR-induced oxidative and inflammatory alterations; however, the present data do not establish a specific molecular mechanism.

#### 2.2.2. Effects of AME on ER Stress Signaling

**Figure 4:**
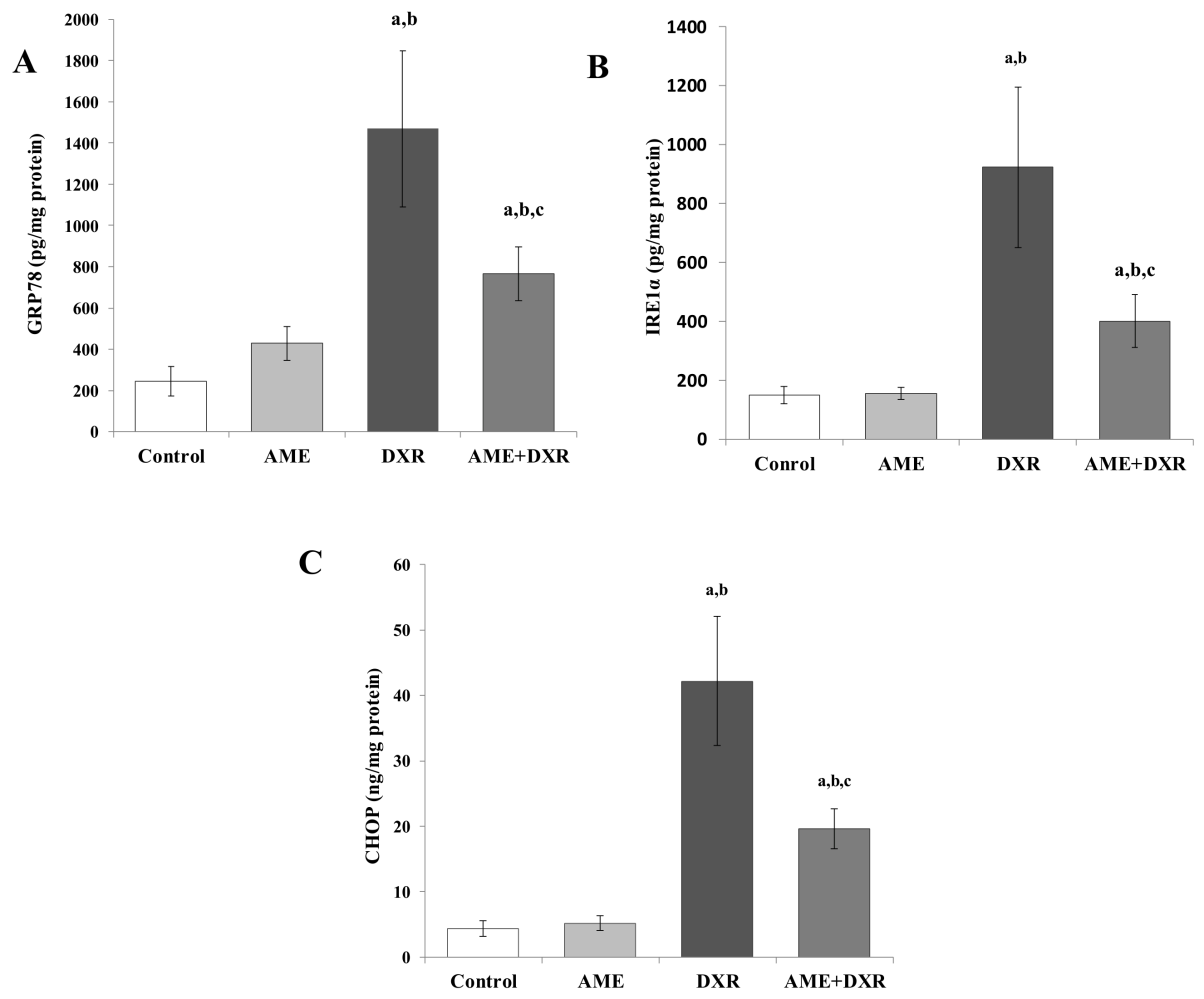
Endoplasmic reticulum stress (ERS) markers in kidney tissues of the experimental groups. (**A**) 78 kDa glucose-regulated protein (GRP78), (**B**) inositol-requiring enzyme-1α (IRE1α), and (**C**) C/EBP homologous protein (CHOP). Data are expressed as mean ± standard deviation (SD) (*n* = 9). Statistical analysis was performed using one-way analysis of variance (ANOVA) followed by Tukey’s post hoc test. Differences were considered statistically significant at *p* < 0.05. a: significantly different from the Control group; b: significantly different from the AME group; c: significantly different from the DXR group. AME: *Aronia melanocarpa* extract; DXR: Doxorubicin.

As shown in Figure 4, DXR administration significantly increased renal levels of the ERS markers GRP78, IRE1α, and CHOP compared with the control group (*p* < 0.001), indicating marked ERS activation following DXR exposure. In contrast, AME pretreatment significantly reduced the levels of these specific ERS indicators (GRP78, IRE1α, and CHOP) compared with the DXR group (*p* < 0.001).

ER stress is a recognized mechanism in chemotherapy-induced nephrotoxicity: whereas transient UPR activation is adaptive, sustained ER stress can shift the UPR toward CHOP-mediated apoptosis and tubular injury [12,13]. In the present study, DXR administration increased renal GRP78, IRE1α, and CHOP levels, and AME pretreatment attenuated these changes, whereas the PERK and ATF6 branches were not evaluated. Because these ER stress markers changed in parallel with redox and apoptotic parameters, the results are consistent with a possible link between oxidative stress, ER stress, and apoptosis during DXR-induced renal injury [11]. Together with the preserved Nrf2 levels in the AME + DXR group, these biomarker-based observations suggest an associative connection rather than definitive evidence of direct Nrf2/ER stress pathway modulation, and they reflect specific changes within the GRP78/IRE1α/CHOP axis rather than a general modulation of the UPR pathway.

#### 2.2.3. Effects of AME on Apoptosis

**Figure 5:**
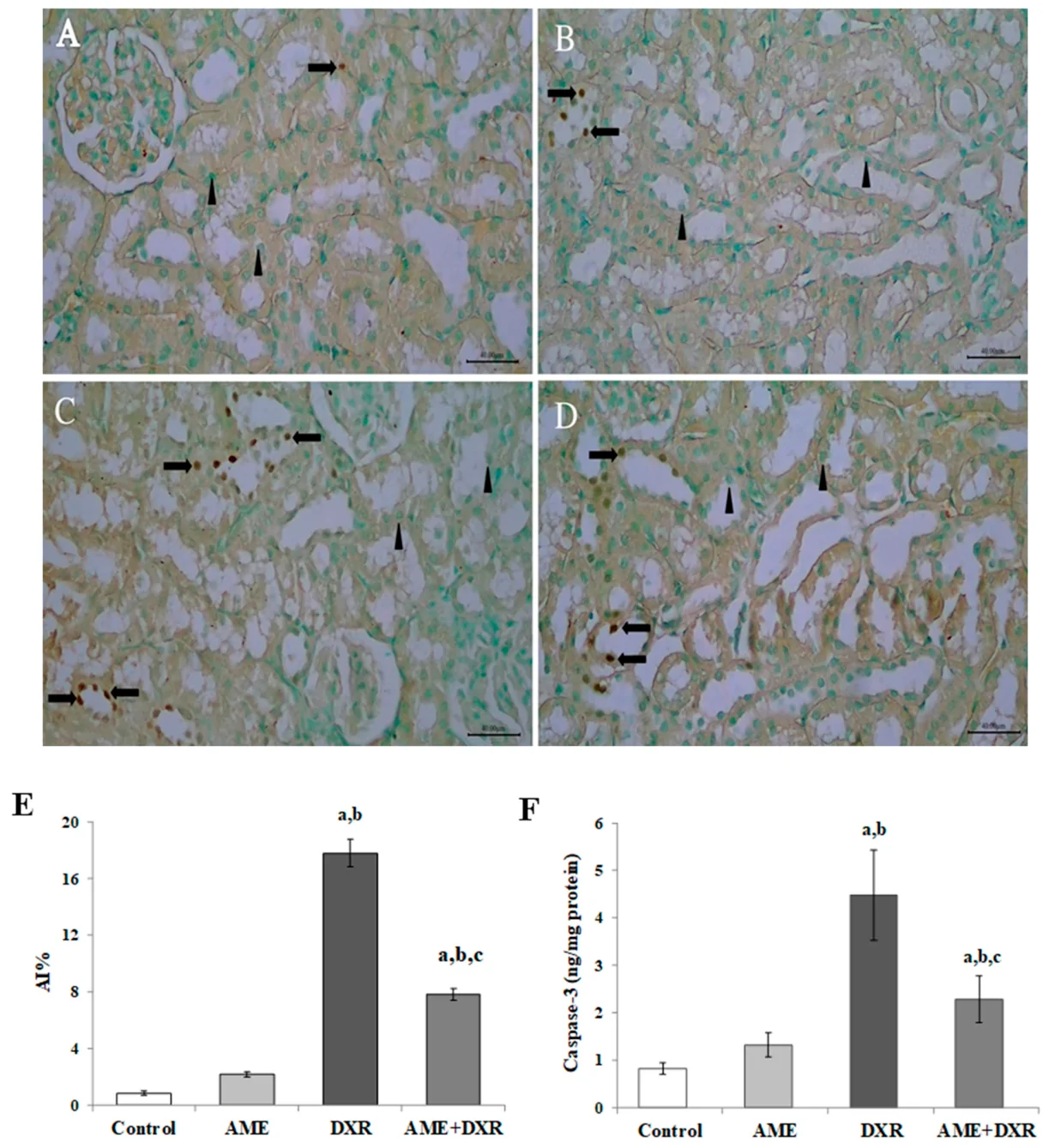
TUNEL-stained photomicrographs of kidney tissues (TUNEL × 400) and apoptosis-related parameters. (**A**) Control group, (**B**) AME group, (**C**) DXR group, and (**D**) AME + DXR group. TUNEL-positive tubular epithelial cells are indicated by arrows, whereas arrowheads indicate TUNEL-negative tubular epithelial cells. (**E**) Apoptotic index (AI%). (**F**) Caspase-3 levels in kidney tissues. Data are expressed as mean ± standard deviation (SD) (*n* = 9). Statistical analysis was performed using one-way analysis of variance (ANOVA) followed by Tukey’s post hoc test. Differences were considered statistically significant at *p* < 0.05. a: significantly different from the Control group; b: significantly different from the AME group; c: significantly different from the DXR group. TUNEL: Terminal deoxynucleotidyl transferase dUTP nick end labeling; AME: *Aronia melanocarpa* extract; DXR: Doxorubicin.

Terminal deoxyribonucleotidyl transferase dUTP nick-end labeling (TUNEL) staining was performed to assess apoptotic cell death in renal tissue (Figure 5). Only a few TUNEL-positive cells were detected in the renal epithelial tissue of the control and AME groups, indicating minimal basal apoptosis (Figure 5A,B). In contrast, DXR administration markedly increased apoptotic cell numbers in both proximal and distal tubular epithelium (Figure 5C). However, AME pretreatment significantly reduced the number of TUNEL-positive cells compared with the DXR group (Figure 5D). Consistent with these histological observations, both the apoptotic index (AI) and caspase-3 levels were significantly elevated in the DXR group but were markedly decreased following AME pretreatment (Figure 5E,F), indicating effective attenuation of apoptosis.

In the present study, AME pretreatment reduced the number of TUNEL-positive tubular cells, the apoptotic index, and caspase-3 levels, and these changes coincided with reduced GRP78, IRE1α, and CHOP levels. Consistent with previous reports that persistent UPR activation can promote CHOP-mediated apoptotic signaling [12], these parallel changes suggest that attenuation of ER stress may contribute to the anti-apoptotic effects of AME. Together with the higher Nrf2 levels observed in the AME + DXR group, these findings are consistent with a possible interplay between redox homeostasis, ER stress regulation, and apoptotic signaling.

Notably, independent experimental studies have reported that chlorogenic acid [33], rutin [34], quercetin [35], protocatechuic acid [36], and C3Ga [15], the major phenolic constituents of AME, each alleviate renal damage through effects attributed to the suppression of oxidative stress, ERS, and apoptosis. Therefore, the abundance of these bioactive compounds in AME is consistent with the simultaneous improvements in redox status, ER stress markers, and apoptosis observed in this study, and provides a plausible compositional basis for the extract’s renoprotective potential. However, since these observations are correlational in nature and individual components were not tested separately, the precise contribution of each compound needs to be experimentally established.

## 3. Materials and Methods

### 3.1. Samples and Extraction

Fresh *A. melanocarpa* berries used in this study were harvested from a cultivated commercial orchard in Trabzon, Turkey. The certified plant material was obtained from a producer registered with the Akçaabat Chamber of Agriculture in October 2023, which guarantees the botanical identity of the cultivar. The berries were stored at −18 °C until processing. A total of 250 g of the fruit was washed with distilled water, carefully blotted dry, and homogenized in 400 mL of 50% (*v*/*v*) aqueous ethanol using a blender. The homogenate was sonicated for 2 h and subsequently macerated on a mechanical shaker for 24 h at room temperature. The mixture was then filtered to completely remove solid residues [37]. The solvent of the extract was completely evaporated under reduced pressure to gravimetrically determine the dry matter content. The working solution was then prepared by re-dissolving the dried extract in 50% (*v*/*v*) aqueous ethanol to achieve a final concentration of 200 mg dry extract/mL prior to administration to the experimental animals.

### 3.2. Total Phenolic and Flavonoid Content of the Sample

The total phenolic content (TPC) of the AME was measured spectrophotometrically using the Folin–Ciocalteu method [38]. In this assay, the intensity of the blue color formed by the reduced product is measured at an absorbance of 760 nm. Gallic acid, a standard phenolic compound, was employed in the preparation of the calibration curve. The TPC was expressed as mg gallic acid equivalents (GAE) per mL of extract.

The extract’s total flavonoid content (TFC) was measured using the aluminum nitrate colorimetric method [39] with minor modifications [40]. Quercetin was used as the reference standard. The absorbance of each standard solution was measured at 414 nm. The TFC of the extract was calculated based on the calibration curve and expressed as mg quercetin equivalents (QE)/g FW and mg QE/mL extract.

### 3.3. Determination of Antioxidant Activity

Total antioxidant activity of the extract was measured by means of the ferric reducing antioxidant power (FRAP) assay [41]. Briefly, FRAP working reagent was prepared by combining 25 mL of 0.3 M acetate buffer (pH 3.6), 2.5 mL of 10 mM TPTZ dissolved in 40 mM HCl, and 2.5 mL of 20 mM FeCl_3_·6H_2_O solution. A 100 µL aliquot of the extract was mixed with 3 mL of the freshly prepared reagent and incubated at 37 °C for 4 min. Absorbance was measured at 595 nm, and the results were expressed as µmol FeSO_4_·7H_2_O/100 g of extract.

DPPH (2,2-diphenyl-1-picrylhydrazyl) radical scavenging activity was measured using a spectrophotometric assay [42]. Briefly, 1.0 mL of 40 µg/mL DPPH radical solution was mixed with 1.0 mL of the extract. The mixture was incubated in the dark for 45 min at 25 °C, and absorbance was measured at 517 nm. In order to calculate the SC_50_ value, samples were prepared by producing six different dilutions of the extract, which were then treated with DPPH solution. The concentration of extract required to scavenge 50% of the DPPH radicals was determined from the graph and expressed as SC_50_ in mg/mL.

### 3.4. Phenolic Composition Analysis Using HPLC-PDA

Prior to HPLC-PDA analysis, phenolic compounds were enriched using a liquid–liquid extraction procedure described previously [43]. Approximately 10 mL of the extract was evaporated to dryness under vacuum at 40 °C to completely remove the alcohol. The residue was then dissolved in 10 mL of distilled water, and the pH was adjusted to 2 using concentrated HCl. Sequential liquid–liquid extraction was performed in triplicate using diethyl ether and ethyl acetate, and the combined organic phases were collected. Following complete removal of solvents, the remaining residue was dissolved in 2 mL of methanol and filtered through a 0.45 µm RC membrane filter prior to injection into the HPLC-PDA system for phenolic compound analysis. Analysis of phenolic composition was conducted using a previously validated method based on 26 phenolic standards [43]. The analysis was performed on a reverse-phase C18 column (250 mm × 4.6 mm, 5 µm; Fortis) connected to an RP-HPLC system (Shimadzu LC-20AT, Kyoto, Japan) equipped with a photodiode array (PDA) detector. A programmed gradient was applied using a mobile phase composed of (A) 2% acetic acid in water and (B) acetonitrile:water (70:30). The injection volume for both samples and standards was 20 µL, the column temperature was maintained at 30 °C, and the flow rate was set at 1.0 mL/min.

### 3.5. Determination of Anthocyanidin via HPLC-PDA

The anthocyanidin content of the extracts was determined using a modified HPLC-PDA as previously described [44]. A C18 column (4.6 × 150 mm, 5 µm; Thermo Fisher Scientific, Waltham, MA,USA) was used for chromatographic separation. A Shimadzu LC-20AT HPLC system (Shimadzu Corporation, Kyoto, Japan) equipped with a PDA detector was employed for the analysis. 

The mobile phase consisted of 100% methanol (A) and 0.5% formic acid (B). The gradient elution program was: 0 min, 86% B; 20 min, 80% B; 23 min, 76% B; 30 min, 0% B; 32 min, 0% B; 33 min, 86% B, with analysis being completed at 35 min. Analytical conditions were optimized as follows: detection wavelength 520 nm, flow rate 1.0 mL/min, injection volume 20 µL, and column temperature 40 °C. Samples and standard solutions were prepared in methanol containing 0.1% formic acid. Calibration curves were produced for cyanidin-3-galactoside, cyanidin-3-glucoside, and cyanidin-3-arabinoside within a concentration range of 3.125–100 ppm. The limit of detection (LOD) and limit of quantification (LOQ) were calculated using the slope (S) and the standard deviation of the intercept (σ) of the calibration curve and defined as LOD = 3.3 × (σ/S) and LOQ = 10 × (σ/S), respectively. Anthocyanidin contents were expressed as µg/g fresh weight (FW).

### 3.6. Animals and Study Design

This study was approved by the Karadeniz Technical University Animal Care and Ethics Committee (Approval No. 2023/46), and all procedures followed the relevant institutional guidelines for the care and use of laboratory animals. Rats were obtained from the KTU Surgical Practice and Research Center and housed at 22 ± 2 °C under a 12 h light/dark cycle with ad libitum access to standard rat chow and water. All animals were monitored daily for general health status, signs of clinical distress, food and water consumption, and body weight. Animals showing severe or persistent distress or marked deterioration would have been euthanized before the scheduled end of the experiment; no animals met these humane endpoint criteria. No expected or unexpected adverse events were observed, no animal died before the scheduled end of the experiment, and no serious adverse events occurred. As no surgery was performed, no perioperative analgesia was required; measures to minimize pain, suffering, and distress were limited to a minimal gavage volume and to the general anaesthesia used for blood sampling and euthanasia. Thirty-six female Sprague–Dawley rats (180–220 g) were randomly assigned to four groups of nine animals each:

Control: Saline was administered by oral gavage for 21 consecutive days.

AME: AME (200 mg/kg body weight/day) was given by oral gavage for 21 consecutive days.

DXR: saline by oral gavage for 21 consecutive days, then a single intraperitoneal injection of DXR (20 mg/kg) on day 21.

AME + DXR: AME (200 mg/kg body weight/day) was given by oral gavage for 21 consecutive days, then a single intraperitoneal injection of DXR (20 mg/kg) on day 21.

Sample-size planning was informed by previous studies on doxorubicin-induced renal injury in rats, in which renal morphological, oxidative stress, apoptotic, and inflammatory alterations were detectable at group sizes comparable to those used here [45]. A formal a priori calculation was not performed; instead, a G*Power sensitivity analysis (version 3.1.9.2; Heinrich Heine University Düsseldorf, Düsseldorf, Germany) showed that a four-group, fixed-effects, omnibus one-way ANOVA with 36 animals (*n* = 9 per group) at α = 0.05 and 90% power would detect a large omnibus effect (Cohen’s f = 0.67). Equal group sizes were used because they give the greatest statistical efficiency for a given total number of animals, in line with the Reduction principle of the 3Rs [46].

Acute nephrotoxicity was induced by a single intraperitoneal injection of DXR (20 mg/kg), a dose previously reported to induce acute renal injury in rats [47,48]. The AME dose (200 mg/kg body weight/day) was based on previous in vivo studies reporting safety and bioactivity at comparable oral doses [49,50,51]. The extract was administered in 50% ethanol (*v*/*v*) by oral gavage at a minimal volume to optimise phenolic solubilisation while keeping ethanol exposure well below recognised systemic toxicity thresholds. A vehicle control group receiving 50% (*v*/*v*) aqueous ethanol alone was not included, in accordance with institutional Animal Ethics Committee recommendations and the Reduction principle, which aims to avoid unnecessary animal use. The 21-day pretreatment regimen ensured sustained exposure to the polyphenolic constituents before the nephrotoxic insult; the present study therefore evaluates the protective potential of AME rather than a therapeutic effect after established injury.

Twenty-four hours after DXR administration, cardiac blood was collected under general anaesthesia with ketamine (60 mg/kg, Vem Pharmaceuticals, Ankara, Turkey) and xylazine (10 mg/kg, Bayer Global, Leverkusen, Germany). Rats were then euthanised by cervical dislocation under deep anaesthesia, and left renal tissues were divided longitudinally into two equal parts: one stored at −80 °C for biochemical analysis and one fixed in 10% formalin for histology. Blood was collected into serum separator tubes, centrifuged at 1800× *g* for 15 min, and the sera stored at −80 °C until analysis.

### 3.7. Histopathological Analysis

The renal tissues were fixed in 10% neutral buffered formaldehyde solution for 48 h. Following fixation, the samples were dehydrated through a graded ethanol series, embedded in paraffin blocks, and sectioned at a thickness of 5 μm. For the purpose of general histopathological evaluation, the sections were stained with hematoxylin and eosin, while the basement membranes were visualized using PAS staining. The stained renal sections were examined under an Olympus BX51 light microscope (Tokyo, Japan). All histological evaluations were performed by two experienced histologists blinded to the experimental groups. Particular attention was paid to the renal cortex and outer medullary regions. Evaluations were conducted in five randomly selected high-power fields (×200 magnification). The PAS staining characteristics of the tubular basement membranes were evaluated qualitatively in each section by assessing the staining of at least 100 tubules at ×400 magnification.

Histopathological scoring was performed semi-quantitatively based on the following criteria: glomerular degeneration, dilatation of Bowman’s space, tubular cell degeneration, intertubular inflammatory cell infiltration, intertubular vascular congestion, and intratubular hyaline accumulation. Each parameter was scored on a scale of 0–3 (0 = no damage; 1 = mild damage, <25%, focal and minimal changes; 2 = moderate damage, 25–50%, multifocal and apparent changes; 3 = severe damage, >50%, diffuse and extensive changes) [52].

#### Detection of Apoptotic Cells by TUNEL Staining

Apoptotic cells were detected using a TUNEL assay kit (HRP-DAB, ab206386, Abcam, Cambridge, MA, USA).Following deparaffinization and rehydration, the staining procedure was conducted in compliance with the manufacturer’s instructions. Following DAB chromogen staining, the sections were counterstained with methyl green. Tubular epithelial cells exhibiting brown-stained nuclei were regarded as TUNEL-positive (DNA fragmentation), while those with green-stained nuclei were considered TUNEL-negative (non-apoptotic). Evaluations were conducted in five randomly selected high-power fields (×400 magnification). The percentage of TUNEL-positive cells was calculated using the following formula [53]:

Percentage of TUNEL (+) cells = TUNEL (+) cell number∕total cell number × 100

### 3.8. Determination of Biochemical Markers

All ELISA and spectrophotometric measurements of renal tissue homogenates and statistical analyses were performed on numerically coded samples by an investigator who was unaware of the group allocation; the codes were revealed only after all measurements had been completed. Levels of serum blood urea nitrogen (BUN) and creatinine (CRE), calculated as renal function tests, were measured using a Beckman Coulter Autoanalyzer (AU5800, Beckman Coulter, Brea, CA, USA) The results were expressed as mg/dL.

The renal tissue samples were homogenized in 1 mL of phosphate-buffered saline (PBS) at 9500 rpm using a homogenizer (IKA T25 Ultra-Turrax, Staufen, Germany). Protein concentrations in the tissue samples were determined using a commercially available kit (Pierce BCA Protein Assay Kit; Thermo Scientific, Rockford, IL, USA) in line with the manufacturer’s instructions. Absorbance was measured at 562 nm using a microplate reader (Versa Max, Molecular Devices, Sunnyvale, CA, USA). All biochemical parameters in the supernatants were expressed as mg of protein. The levels of KIM-1, NGAL, antioxidant-related markers (GSH, SOD1, and Nrf2), and TNF-α were measured in renal tissue homogenates using enzyme-linked immunosorbent assay (ELISA) kits (BT Lab, Shanghai, Zhejiang, China) according to the manufacturer’s instructions. The results were expressed as ng/mg protein.

The level of the lipid peroxidation marker MDA in renal homogenates was determined spectrophotometrically using the thiobarbituric acid reactive substances (TBARS) method [54]. For this purpose, 100 mg of tissue was homogenized in 1 mL of 1.15% KCl and 0.05% Triton X-100 solution and then centrifuged at 3200× *g* for 10 min. The absorbance of the samples and standards (tetramethoxypropane) was measured at 532 nm, and tissue MDA levels were expressed as nmol/mg of protein.

#### Determination of Renal ERS Markers and Apoptosis

The levels of tissue ERS markers (GRP78, IRE1α and CHOP) and caspase-3 were determined using commercial ready-to-use ELISA kits (BT Lab, Zhejiang, China) in compliance with the manufacturer’s recommendations. The absorbances were measured at 450 nm using a microplate reader (Molecular Devices Versamax, Sunnyvale, CA, USA). GRP78 and IRE1α levels were expressed as pg/mg protein, and caspase-3 and CHOP as ng/mg protein.

### 3.9. Statistical Analysis

All statistical analyses were carried out using IBM SPSS Statistics for Windows, version 23.0 software (IBM Corp., Armonk, NY, USA). The Shapiro–Wilk test was applied to assess the normality of data distribution. Variables exhibiting normal distribution were expressed as mean ± standard deviation (SD). Comparisons among more than two groups were conducted using one-way analysis of variance (ANOVA). When a significant difference was observed, Tukey’s post hoc multiple comparison test was applied to determine the specific group differences. A *p*-value < 0.05 was considered statistically significant.

## 4. Conclusions

AME pretreatment showed partial protective effects against DXR-induced renal injury in this acute experimental model, as evidenced by improvements in renal injury markers and histopathological alterations, together with attenuation of oxidative stress, inflammatory responses, ER stress-related markers, and apoptosis. Complementing with the chemical characterization of the extract, these findings support the nephroprotective potential of phenolic-rich AME as a whole. The concurrent changes in Nrf2 levels and ER stress-related biomarkers provide additional insight into the cellular responses associated with AME pretreatment; however, these findings are correlational and do not establish a causal role of Nrf2 or of specific ER stress signaling pathways. Overall, the consistent biochemical and histopathological findings support a protective effect of AME against acute DXR-induced renal injury, although protection was partial, as several parameters did not return to control levels.

Nevertheless, this study has several limitations. First, the study was conducted using an acute experimental model with a single AME dose and a single post-DXR evaluation time point, which limits the assessment of dose-dependent and long-term effects. Second, ER stress was evaluated only through GRP78, IRE1α, and CHOP, whereas other important UPR branches, including PERK and ATF6, were not assessed. Third, the assessment of signaling-related markers was limited to protein levels measured by ELISA, and gene expression analyses could not be performed because of budget constraints. These measurements do not establish pathway activation or causal relationships; therefore, the observed changes in Nrf2 and ER stress-related markers should be interpreted as associative. Fourth, the individual contributions of specific AME constituents, including C3Ga and chlorogenic acid, were not experimentally evaluated; therefore, the observed protective effects cannot be attributed to any individual constituent. Fifth, AME was administered in 50% (v/v) aqueous ethanol without a dedicated vehicle control group; therefore, a potential influence of the solvent cannot be excluded. Finally, the potential effect of AME on the anticancer efficacy of DXR was not investigated. Further studies incorporating multiple doses and time points, gene expression and functional analyses, individual constituents, appropriate vehicle controls, and tumor-bearing models are needed to address these limitations.

## Figures and Tables

**Table 1:** Phenolic content and antioxidant activity values of the *A. melanocarpa* extract.

Parameters	Unit (Fruit)	Results In Fruit	Unit (Extract)	Results In Extract
Total Phenolic content (TPC)	mg GAE/g FW	15.75 ± 1.04	mg GAE/mL extract	3.11 ± 0.21
Total Flavonoid content (TFC)	mg QE/g FW	1.16 ± 0.03	mg QE/mL extract	0.23 ± 0.01
Total antioxidant capacity (FRAP)	µmol FeSO_4_·7H_2_O/g FW	191.93 ± 3.07	µmol FeSO_4_·7H_2_O/mL extract	37.95 ± 0.61
DPPH radical scavenging	(SC_50_) mg/mL	0.23 ± 0.04	(SC_50_) mg/mL	0.23 ± 0.04
Anthocyanin	µg/g FW		µg/mL extract	
Cyanidin-3-Galactoside	“ ”	2440	“ ”	812
Cyanidin-3-Glucoside	“ ”	<LOD	“ ”	<LOD
Cyanidin-3-Arabinoside	“ ”	350	“ ”	117
Phenolic acids	µg phenolic/g FW		µg phenolic/mL extract	
Protocatechuic acid	“ ”	25.32	“ ”	5.01
Chlorogenic acid	“ ”	251.83	“ ”	49.79
*t*-cinnamic acid	“ ”	0.24	“ ”	0.05
*p*-Coumaric acid	“ ”	3.06	“ ”	0.61
Caffeic acid	“ ”	1.50	“ ”	0.30
Flavonoids	µg phenolic/g FW		µg phenolic/mL extract	
Quercetin		7.51	“ ”	1.48
Rutin	“ ”	32.29	“ ”	6.39
Chrysin	“ ”	0.53	“ ”	0.10
Pinocembrin	“ ”	0.45	“ ”	0.09

**Table 2:** Histopathological grading of renal alterations among the experimental models.

Parameters	Control	AME	DXR	AME + DXR	*p*
Dilatation of Bowman’s space	0.17 ± 0.16	0.50 ± 0.22	2.17 ± 0.31 ^a,b^	0.67 ± 0.21 ^c^	0.0001
Glomerular degeneration	0.33 ± 0.56	0.50 ± 0.20	2.18 ± 0.17 ^a,b^	0.66 ± 0.21 ^c^	0.0001
Tubular epithelial degeneration	0.30 ± 0.21	0.83 ± 0.17	2.33 ± 0.21 ^a,b^	1.50 ± 0.22 ^a,c^	0.0001
Intertubular inflammatory cell infiltration	0.18 ± 0.17	0.33 ± 0.21	0.70 ± 0.22 ^a,b^	0.42 ± 0.22 ^a,c^	0.038
Intertubular vascular congestion	0.50 ± 0.22	0.83 ± 0.17	0.68 ± 0.21	0.60 ± 0.21	0.724
Intratubular hyaline accumulation	0.17 ± 0.11	0.67 ± 0.21	2.17 ± 0.31 ^a,b^	1.33 ± 0.21 ^a,c^	0.0001

Data are expressed as mean ± SD (*n* = 9). Statistical analysis was performed using one-way analysis of variance (ANOVA) followed by Tukey’s post hoc test. Differences were considered statistically significant at *p* < 0.05. a: significantly different from the Control group; b: significantly different from the AME group; c: significantly different from the DXR group.

**Table 3:** Modulatory effects of AME on serum biochemical parameters and renal injury biomarkers.

Parameters	Control	AME	DXR	AME + DXR
KIM-1 (ng/mg protein)	0.71 ± 0.16	1.64 ± 0.45	5.83 ± 0.64 ^a,b^	3.18 ± 0.81 ^a,b,c^
NGAL (ng/mg protein)	4.0 ± 1.19	8.0 ± 0.65	23.3 ± 4.17 ^a,b^	15.1 ± 1.10 ^a,b,c^
BUN (mg/dL)	16.7 ± 3.17	19.2 ± 1.33	26.0 ± 2.17 ^a,b^	21.2 ± 1.47 ^a,c^
CRE (mg/dL)	0.28 ± 0.08	0.35 ± 0.05	0.42 ± 0.05 ^a,b^	0.37 ± 0.03 ^a,c^

Data are expressed as mean ± SD (*n* = 9). Statistical analysis was performed using one-way analysis of variance (ANOVA) followed by Tukey’s post hoc test. Differences were considered statistically significant at *p* < 0.05. a: significantly different from the Control group; b: significantly different from the AME group; c: significantly different from the DXR group.

## Data Availability

The data supporting the findings of this study are available from the corresponding author upon reasonable request.

## Supplementary Materials

The following supporting information can be downloaded at: https://www.mdpi.com/article/10.3390/molecules31203732/s1, Figure S1, HPLC–PDA chromatogram of 26 phenolic standards; Figure S2, HPLC–PDA chromatograms of 3 anthocyanin standards.

## Abbreviations

The following abbreviations are used in this manuscript:

AME	*A. melanocarpa* extract
DXR	doxorubicin
ERS	endoplasmic reticulum stress
BUN	blood urea nitrogen
KIM-1	renal injury molecule
NGAL	neutrophil gelatinase-associated lipocalin
MDA	malondialdehyde
GSH	glutathione
SOD1	superoxide dismutase
Nrf2	nuclear factor erythroid 2–related factor
TNF-α	tumor necrosis factor alpha
CHOP	C/EBP homologous protein
GRP78	glucose-regulated protein 78
IRE1α	inositol-requiring enzyme-1 alpha
UPR	unfolded protein response
ROS	reactive oxygen species
TPC	total phenolic content
TFC	total flavonoid content
FRAP	ferric reducing antioxidant power
DPPH	2,2-Diphenyl-1-picrylhydrazyl
SC_50_	half-maximal scavenging concentration
ANOVA	analysis of variance.
