# Supplementary material for: Phenolic-Rich Aronia melanocarpa (cv. Nero) Extract Attenuates Doxorubicin-Induced Nephrotoxicity Associated with Reduced Oxidative Stress, Inflammation, and Endoplasmic Reticulum Stress
Source: Molecules. 2026 Oct 17;31(20):3732. doi: 10.3390/molecules31203732 (PMC13707431; doi:10.3390/molecules31203732)
Supplement: Supplementary file 1 [file molecules-31-03732-s001.zip › Figure S1.pdf]

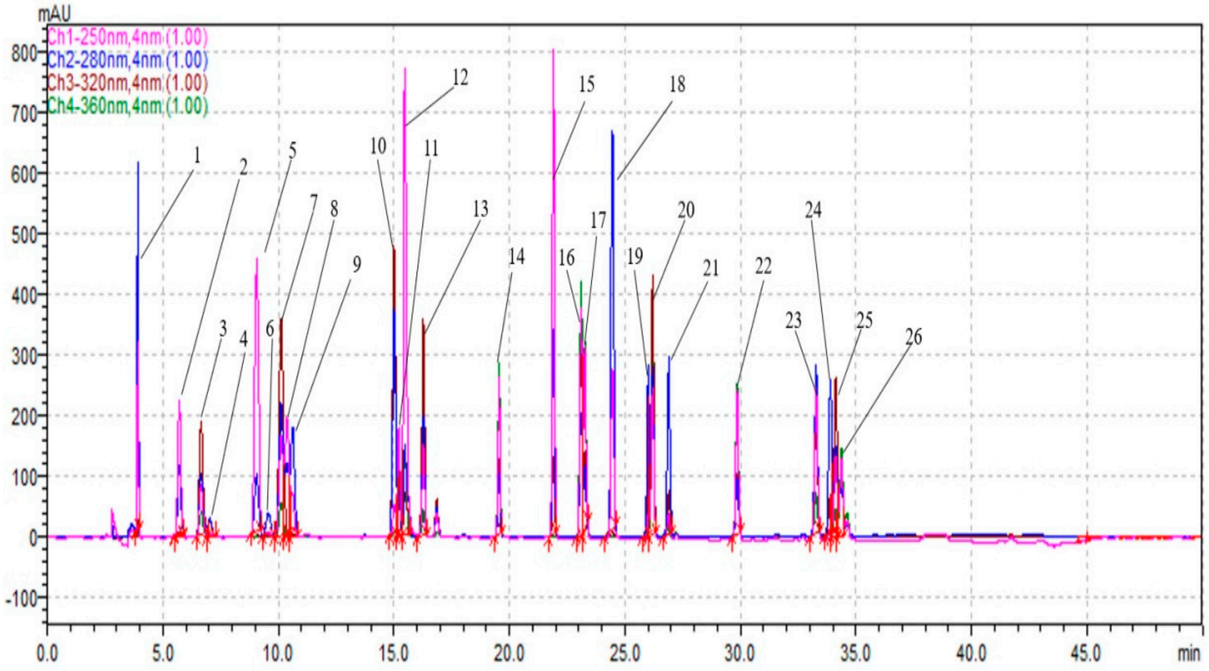

Supplement (Figure S1). Phenolic Standard Chromatogram. 1. Gallic acid, 2. Protocatechuic acid, 3. Chlorogenic acid, 4. Catechin hydrate, 5. p-OH Benzoic acid, 6. Epicatechin, 7. Caffeic acid, 8. Vanillic acid, 9. Syringic acid, 10. p-Coumaric acid, 11. Rutin, 12. Ellagic acid, 13. Ferulic acid, 14. Myricetin, 15. Daidzein, 16. Luteolin, 17. Quercetin, 18. t-Cinnamic acid, 19. Naringenin, 20. Apigenin, 21. Hesperetin, 22. Rhamnetin, 23. Chrysin, 24. Pinocembrin, 25. CAPE, 26. Galangin
