# Supplementary material for: Phenolic-Rich Aronia melanocarpa (cv. Nero) Extract Attenuates Doxorubicin-Induced Nephrotoxicity Associated with Reduced Oxidative Stress, Inflammation, and Endoplasmic Reticulum Stress
Source: Molecules. 2026 Oct 17;31(20):3732. doi: 10.3390/molecules31203732 (PMC13707431; doi:10.3390/molecules31203732)
Supplement: Supplementary file 1 [file molecules-31-03732-s001.zip › Figure S2.pdf]

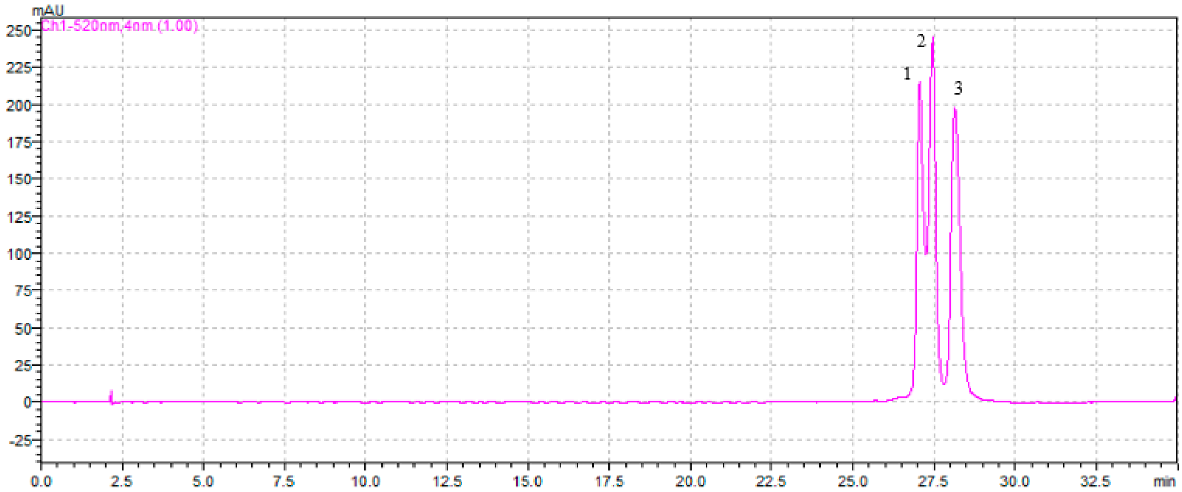

Figure S2. Representative HPLC-PDA chromatograms of anthocyanin standards: (1) cyanidin-3-galactoside (LOD: 0.074 mg/mL, LOQ: 0.223 mg/mL), (2) cyanidin-3-glucoside (LOD: 0.308 mg/mL, LOQ: 0.934 mg/mL), and (3) cyanidin-3-arabinoside (LOD: 0.135 mg/mL, LOQ: 0.408 mg/mL).
